# Evaluating vision-related quality of life in preoperative age-related cataract patients and analyzing its influencing factors in China: a cross-sectional study

**DOI:** 10.1186/s12886-015-0150-8

**Published:** 2015-11-07

**Authors:** Min Zhu, Jiaming Yu, Jinsong Zhang, Qichang Yan, Yang Liu

**Affiliations:** School of Public Health, China Medical University, Shenyang, 110122 China; Department of Ophthalmology, The Fourth Affiliated Hospital of China Medical University, Shenyang, 110001 China

**Keywords:** Cataract, Quality of life, Visual function, NEI-VFQ-25, China

## Abstract

**Background:**

To evaluate vision-related quality of life in preoperative age-related cataract patients in China, using the Chinese version of the National Eye Institute Visual Function Questionnaire-25 (CHI-NEI-VFQ-25), together with analyses of its influencing factors.

**Methods:**

Cataract patients were interviewed using the CHI-NEI-VFQ-25, and their demographic information was recorded. The Cronbach α coefficient was used to determine the internal consistency of the CHI-NEI-VFQ-25. Multi-trait analyses were used to assess construct validity, including item convergent validity and item discriminant validity. The data were evaluated by descriptive statistical analyses, by the Kruskal–Wallis rank sum test, and by multinomial logistic regression.

**Results:**

The Cronbach α coefficients were all above 0.8, except for the driving subscale. All items passed the convergent and discriminant validity tests. The composite score was 63.0. The lowest five subscale scores were in general vision (40.0), mental health (37.5), role difficulty (37.5), near vision activities (50.0), and dependence (58.3). Except for the subscale scores of general health and ocular pain, the composite scores and subscale scores were positively associated with the best-corrected visual acuity (BCVA) in the better eye. Multinomial logistic regression showed that sex, age, and educational attainment were significantly associated with the composite score and subscale scores.

**Conclusions:**

BCVA in the better eye was the most important determinant associated with the decrease in vision-related quality of life. Visual impairment had more impact on the psychosocial parameter than on the other parameters of the patients’ quality of life. Among all the demographic characteristics, including sex, age, and educational attainment, influenced the quality of life in age-related cataract patients.

## Background

Cataracts are the leading cause of visual disability in China, accounting for over 60 % of these disabilities [1]. Cataracts are primarily an age-related disease. Of those with visual disability due to cataracts, 90.9 % are aged 60 years or older [1]. Compared with other common age-related conditions, visual impairment has greater impact on the quality of life of older adults, including increased difficulties in daily activities [2, 3], increased depression and social isolation, and an increased risk of falls and fractures [4–7]. However, little is presently known about the impact of visual impairments on the quality of life of Chinese age-related cataract patients.

The National Eye Institute Visual Function Questionnaire-25 (NEI-VFQ-25) is a reliable questionnaire used to evaluate vision-related quality of life. The original NEI-VFQ-25, which was a shortened version of NEI-VFQ-51, was developed by Mangione and co-workers [8], and was widely used by many countries throughout the world [9–13]. The Chinese version of NEI-VFQ-25 (CHI-NEI-VFQ-25) was developed by Chan and co-workers in Hong Kong [14]. They showed that this questionnaire was an effective means in assessing the vision-related quality of life in Chinese patients with eye diseases [14]. The purpose of our study was therefore to evaluate the vision-related quality of life in preoperative age-related cataract patients in mainland China, using the CHI-NEI-VFQ-25, together with identifying its influencing factors. The results will help the Chinese public understand the impact of age-related cataracts on the quality of life in China, so that they will have more impetus to maintain their visual health.

## Methods

### Population

A cross-sectional survey was conducted between May 2011 and December 2012 in the Ophthalmology Department of the Fourth Affiliated Hospital of China Medical University, Shenyang, China. All of the preoperative cataract patients who matched the inclusion criteria were recruited for this study. The inclusion criteria were as follows: patients aged 40 years or older; patients diagnosed as age-related cataract(s) without other major eye diseases, such as glaucoma, macular degeneration, or diabetic retinopathy; and patients who had not previously undergone cataract surgery in either eye. Cataract patients were excluded if they could not understand the questions, were unable to communicate, or had other serious systemic diseases that affected their quality of life (e.g., paralysis or mental disorders). All of the cataract patients had a complete ophthalmic and physical examination before the operation. A total of 408 cataract patients were identified as eligible for this survey. Seven cataract patients refused to participate, and 401 eligible patients completed the questionnaire.

This study was approved by the ethics committee of China Medical University. Verbal agreement for informed consent was obtained from all patients. All study procedures adhered to the principles outlined in the Declaration of Helsinki for research involving human subjects.

### Instruments and interview

We used the CHI-NEI-VFQ-25 to assess the quality of life of age-related cataract patients. All patients were evaluated in an interview, using the CHI-NEI-VFQ-25, by the interviewer (Min Zhu) the day before surgery. The interview was conducted in the privacy of a small conference room. The interviewer read each question to the patient and recorded the responses. It took approximately 30 min to complete the questionnaire. Demographic information, including age, sex, occupation, marital status, educational attainment, and household income, were also documented. The CHI-NEI-VFQ-25 consisted of 25 questions, grouped into one question assessing general health, and 11 subscales involving visual difficulties in everyday life, as well as vision-related psychosocial parameters such as mental health, social function, and role difficulties. Each subscale included one or more questions. The original answer to each question was converted to 0–100 points; 100 was the best possible score, and 0 was the worst possible score. The subscale scores were the average of one or more questions. The composite score of the NEI-VFQ-25 was the average of all the questions, except for the question about assessing general health [8]. The higher composite scores indicated a higher visual functionality.

### Visual acuity testing

The best-corrected visual acuity (BCVA) was tested with a “Tumbling E” Eye Chart at a distance of 5 m, and the BCVA was expressed in decimal notations. If the patients could not read the largest optotype on the chart, the test distance was reduced until it was read by the patient. If the patients could not see the largest optotype at a distance of 1 m, further BCVA assessment was performed by finger counting, hand movement, and/or light perception. BCVA in the better eye was used to represent the presenting visual status of the patient in this study. The BCVA was sourced from the patient’s medical records which ensured consistent measurements that were undertaken by doctors. It also further facilitated, on the basis of BCVA, compliance of categorization of visual impairment divisions as specified by the World Health Organization (WHO). Patients were divided into four groups according to the categories of visual impairment established by WHO [15]: blindness, BCVA < 0.05 in the better eye; low vision, 0.05 ≤ BCVA < 0.3 in the better eye; monocular visual impairment, BCVA ≥ 0.3 in the better eye and BCVA < 0.3 in the other eye; and mild visual impairment, BCVA ≥ 0.3 in both eyes. Monocular visual impairment, which is not a WHO definition, was used to identify patients who had normal or near normal vision in the better eye (BCVA ≥ 0.3) and visual impairment in the other eye (BCVA < 0.3) [16].

### Reliability and validity

The Cronbach α coefficient was used to determine the internal consistency of the items. We considered a Cronbach’s α coefficient over 0.7 to represent a reliable scale [8].

Multi-trait analysis was used to assess construct validity, including item convergent validity and item discriminant validity [17]. For each item, if the correlation between the score and the subscale heading score to which that item belonged was 0.4 or higher, that item was validated for convergent validity [17]; if the correlation between the item score and the subscale score to which that item belonged was significantly greater than the correlations between the item score and the scores on all other subscales, the item was validated for discriminant validity [17].

### Statistical analysis

Descriptive statistics were used to determine the basic demographic characteristics of the population. Median and range were used to describe the composite score and the score of each subscale. The Kruskal–Wallis rank sum test was used to compare the composite score and subscale scores among cataract patients with different sociodemographic characteristics and different vision statuses. If the results of Kruskal–Wallis rank sum test showed statistical differences, the Wilcoxon rank sum test was used to perform pairwise comparisons. The pairs tested in the pairwise comparisons included the blindness group and the low vision group, the blindness group and the monocular visual impairment group, the blindness group and the mild visual impairment group, the low vision group and the monocular visual impairment group, the low vision group and the mild visual impairment group, and the monocular visual impairment group and the mild visual impairment group. In order to avoid an increase in the total probability of a type 1 error, the α level for pairwise comparisons was adjusted to 0.017 (0.05/3). Multinomial logistic regression was used to determine potential factors influencing the composite score and subscale scores. All the potential influencing factors were incorporated into the model as categorical variables.

All statistical analyses used the SPSS 12.0 software (SPSS, Chicago, IL, USA). A two-tailed *P* value < 0.05 was considered significant.

## Results

### General characteristics of the samples

There were 401 participants in this survey, including 188 males. The mean age of the patients was 69 ± 10.1 years (mean ± SD). The majority, 81.5 %, of the patients were retired. Regarding marital status, 91.8 % of the patients were married, and the remainder were widowed. Concerning educational attainment, over 50 % of the patients completed upper secondary school education or higher education. An income of 2000–3000 RMB per month was most common. Among the patients, 94.3 % had medical insurance. The mean BCVA in the better eye of the patients was 0.4 (median). According to the WHO categories of visual impairment, 9.7 % of the patients were blind, 22.4 % had low vision, 46.6 % had monocular visual impairment, and 21.2 % had mild visual impairment.

### Reliability and validity

Except for the driving subscale, the Cronbach α coefficients of the multi-item subscales were greater than 0.8, indicating that these subscales had satisfactory internal consistency and reliability. All items passed the convergent and discriminant validity tests (Table 1).

**Table 1:** Reliability and validity of the CHI-NEI-VFQ-25 subscales

Subscale	Number of items	Cronbach α	Convergent validity	Discriminant validity
General health	1	NA	NA	NA
General vision	1	NA	NA	NA
Ocular pain	2	0.872	100	100
Near vision activities	3	0.863	100	100
Distance vision activities	3	0.853	100	100
Social function	2	0.866	100	100
Mental health	4	0.807	100	100
Role difficulty	2	0.982	100	100
Dependence	3	0.886	100	100
Driving	3	0.586	100	100
Color vision	1	NA	NA	NA
Peripheral vision	1	NA	NA	NA

CHI-NEI-VFQ-25, Chinese version of the National Eye Institute Visual Function Questionnaire-25

NA, not applicable for subscales with only one item

### Composite and subscale scores of the CHI-NEI-VFQ-25

As shown in Table 2, the composite score of the CHI-NEI-VFQ-25 was 63.0 for all patients. The lowest five subscale scores were for mental health (37.5), role difficulty (37.5), general vision (40.0), near vision activities (50.0), and dependence (58.3). The near vision activities subscale score was the lowest among the subscale scores for blind patients. Except for the general vision subscale score, the mental health subscale scores were the lowest in the other three groups.

**Table 2:** Composite and subscale scores of the CHI-NEI-VFQ-25 (median range)

Subscale	Scores of different vision statuses				Score of the whole group
	Blindness	Low vision	Monocular visual impairment	Mild visual impairment	
General health	50.0(100.0)	50.0(100.0)	75.0(100.0)	75.0(100.0)	75.0(100.0)
General vision^**^	20.0(40.0)	20.0(60.0)	40.0(60.0)	40.0(40.0)	40.0(60.0)
Ocular pain	100.0(50.0)	100.0(87.5)	100.0(75.0)	100.0(75.0)	100.0(87.5)
Near vision activities^**^	0 (91.7)	41.7(100.0)	58.3(100.0)	58.3(100.0)	50.0(100.0)
Distance vision activities^**^	16.7(91.7)	50.0(100.0)	75.0(100.0)	75.0(100.0)	62.5(100.0)
Social function^**^	25.0(100.0)	68.8(100.0)	87.5(100.0)	87.5(87.5)	87.5(100.0)
Mental health^**^	18.8(81.3)	25.0(81.3)	43.8(81.3)	37.5(81.3)	37.5(81.3)
Role difficulty^**^	12.5(100.0)	25.0(100.0)	50.0(100.0)	50.0(100.0)	37.5(100.0)
Dependence^**^	16.7(75.0)	37.5(75.0)	66.7(83.3)	58.3(75.0)	58.3(83.3)
Color vision^**^	50.0(100.0)	100.0(100.0)	100.0(100.0)	100.0(100.0)	100.0(100.0)
Peripheral vision^**^	25.0(100.0)	50.0(100.0)	75.0(100.0)	75.0(100.0)	75.0(100.0)
Composite score^**^	27.1(76.1)	50.1(78.9)	68.0(76.4)	65.5(75.3)	63.0(85.4)

CHI-NEI-VFQ-25, Chinese version of the National Eye Institute Visual Function Questionnaire-25

The Driving subscale was omitted, because there were too few cases for analysis

^**^The scores in the four vision status groups were significantly different, *P* < 0.01. The Wilcoxon rank sum test was used to perform pairwise comparisons; The median of the composite scores and the medians of the subscale scores between any two vision status groups were significantly different (*P* < 0.017), except for the difference between the monocular visual impairment group and the mild visual impairment group (*P* > 0.05)

Except for the general health and ocular pain subscale scores, the subscale scores and composite scores among the four visual status groups were significantly different (*P* < 0.01). The median composite score for the blind group was 30 lower than that of the low vision group, 40.9 lower than that of the monocular visual impairment group, and 38.4 lower than that of the mild visual impairment group (Table 2). The median composite score in the low vision group was 17.9 lower than the monocular visual impairment group, and 15.4 lower than the mild visual impairment group (Table 2). There were no significant differences in the scores between patients with monocular visual impairment and patients with mild visual impairment (*P* > 0.05).

### Factors influencing the composite and subscale scores

The associations of the subscale scores and composite scores with demographic characteristics are shown in Table 3. The positive influencing factors included educational attainment and household income; the negative influencing factors included age, employment status, and marital status. The sex was positively associated with the subscale score of ocular pain, but negatively associated with the subscale scores of distance vision activities and dependence.

**Table 3:** Relationships of the subscale and composite scores with demographic characteristics

Scores	Sex	Age	Employment status	Marital status	Education attainment	Household income
General health	0	-	-	0	0	0
General vision	0	0	0	0	0	0
Ocular pain	+	0	0	0	0	0
Near vision activities	0	-	0	-	+	+
Distance vision activities	-	-	0	-	+	+
Social function	0	-	0	-	+	+
Mental health	0	0	0	0	+	0
Role difficulty	0	0	0	0	0	0
Dependence	-	-	0	0	+	+
Color vision	0	-	0	0	+	0
Peripheral vision	0	-	0	0	0	0
Composite score	0	-	0	-	+	+

+, positively correlated, *P* < 0.05; 0, not significantly correlated; −, negatively correlated, *P* < 0.05

Multinomial logistic regression was used to select the major demographic factors influencing the composite score and subscale scores (Table 4). As visual acuity was the dominate factor in determining the quality of life, the visual status was also introduced into the regression model as a variable. The variables that had statistical significance were sex, age, educational attainment, and vision status. The subscale score of distance visual activities for males was higher than for females. The composite scores and the subscale score of social function in different age groups decreased from the younger age group to the older age group. An increase in the level of educational attainment was associated with an increase in the subscale score of mental health. Employment status, marital status, and household income had no statistically significant effect on the composite score and subscale scores. Multinomial logistic regression confirmed that the vision status had the greatest effect on the composite score and subscale scores.

**Table 4:** Multinomial logistic regression analyses for the CHI-NEI-VFQ-25 subscale and composite scores

Scores		Sex	Age	Employment status	Marital status	Education attainment	Household income	Vision status
General health	*χ* ^2^	N	12.045	7.188	N	N	N	N
	*P*		0.061	0.126				
Near vision activities	*χ* ^2^	N	7.489	N	2.141	5.484	3.208	50.856
	*P*		0.278		0.343	0.483	0.782	0.000
Distance vision activities	*χ* ^2^	6.463	8.710	N	1.681	4.310	4.926	47.036
	*P*	0.039	0.191		0.431	0.635	0.553	0.000
Social function	*χ* ^2^	N	18.411	N	1.502	11.789	5.022	54.640
	*P*		0.005		0.472	0.067	0.541	0.000
Mental health	*χ* ^2^	N	N	N	N	19.435	N	36.056
	*P*					0.003		0.000
Dependence	*χ* ^2^	3.944	7.043	N	N	4.519	N	68.924
	*P*	0.139	0.317			0.607		0.000
Color vision	*χ* ^2^	N	8.097	N	N	2.936	N	45.852
	*P*		0.231			0.817		0.000
Peripheral vision	*χ* ^2^	N	9.869	N	N	N	N	54.064
	*P*		0.130					0.000
Composite score	*χ* ^2^	N	12.768	N	2.207	6.394	5.128	57.944
	*P*		0.047		0.332	0.381	0.527	0.000

CHI-NEI-VFQ-25, Chinese version of the National Eye Institute Visual Function Questionnaire-25

N: Based on the Kruskal–Wallis rank sum test, the variable had no significant association with the score and was not introduced into the multinomial logistic regression model

## Discussion

The results of this study demonstrated that the internal consistency reliability and construct validity of the CHI-NEI-VFQ-25 were satisfactory in mainland China, except for the driving subscale. The Cronbach α coefficients of the multi-item subscales in our study were similar or better than those of the original Chinese version in the Hong Kong study with the exception of the distance vision activities subscale. The Cronbach α coefficient in the driving subscale, as in the original study, was lower than 0.7 [14]. The excessively high non-response rate of the driving subscale may have caused the lower Cronbach α coefficient in this subscale. Other studies also reported that the driving subscale had the highest non-response rate [11, 12, 18]. As in the Hong Kong study [14], the driving subscale was omitted when the composite score was calculated.

The composite score of Chinese cataract patients (63.0) was lower than those in Taiwan (73.5, *n* = 53) [18] and Japan (66.0, *n* = 96) [11], respectively. It should be noted that the patients who undertook this study were about to undergo cataract surgery which was expected to result in lower visual acuity and lower NEI-VFQ scores. Alternatively, patients in Taiwan and Japan may not have undergone cataract surgery or their visual acuities may not have been seriously impaired [11, 18]. Another study by Labiris et al. assessed the quality of life for cataract patients waiting for cataract surgery in Greece [13]. The composite score of the Greek cataract patients was higher than the Chinese patients with mild visual impairment, whose age (62.5 years, *n* = 85) and BCVA (0.3–0.8 in both eyes) were similar or even better than the visual acuity of the Greek cataract patients. It followed that vision-related quality of life not only correlated with visual acuity, but was also influenced by other factors, such as socioeconomic level, cataract surgical service, and support from family and society.

Cataract significantly decreased vision-related quality of life in most of the measured parameters in the present study. Mental health, role difficulty, general vision, near vision activities, and dependence were the most affected parameters. Because the mental health and role difficulty were classified as psychosocial parameters, the results also indicated that visual impairment had more impact on the psychosocial parameter than the other quality of life parameters. Greater visual impairment was significantly associated with lower subscale scores which in turn predicted increased limitations in daily activities. These limitations can leads to a decrease in social interactions and an increase in dependence further resulting in greater psychosocial symptoms. Fagerström et al. also showed that psychiatric symptoms increased with the deterioration of visual acuity, and diminished when visual acuity improved [19]. These results suggest that more attention should be addressed toward the psychological health of patients who cannot receive cataract surgery in a short period of time, and also suggest that more care, such as low vision aids and counseling, should be provided.

The results of our study also showed that the BCVA in the better eye was the most important determinant associated with a rapid decrease in the vision-related quality of life. Except for the subscale of general health and ocular pain, the subscale scores and composite score of the blind and low vision groups were much lower than the mild visual impairment group. Regression analyses also proved that the BCVA in the better eye was a significant determinant of the quality of life. Chan et al. also reported that the visual acuity in the better eye had a high correlation with the quality of life [20], and the response of patients in our study indicated that the primary cause for the decrease in the quality of life was the reduction or loss of visual acuity. Broman et al. reported that the impact of cataracts on the quality of life was largely mediated through its effect on visual acuity; glaucoma and diabetic retinopathy probably had other effects besides visual acuity that had an impact on the quality of life [9]. Thus, vision rehabilitation through surgery will significantly improve the quality of life for cataract patients.

In addition to visual acuity, the vision-related quality of life was also influenced by other factors. The demographic variables which had statistical significance in the Kruskal–Wallis rank sum test were introduced into the multinomial logistic regression model, and showed that sex, age, and educational attainment played an important role in vision-related quality of life. However, income was significantly associated with the composite score and subscale scores, which was inconsistent with the results of another report [9].

Our findings provided further evidence that visual impairment had a greater impact on the quality of life of older adults, with the composite scores and the subscale scores of social function negatively related with age (*P* < 0.05). It should be noted that patients in the younger age group often play a more active and important societal and familial role than those in the older age group. This could lead to a higher expectation of the visual abilities and may prompt younger adults to seek treatment sooner. This in turn may result in their visual impairment from cataracts being less severe in the early stages thus having a lesser influence on their quality of life. Broman et al. studied the relationship between the NEI-VFQ-25 subscale scores and age, and reported that all of the subscale scores were associated with age, except for general vision and mental health [9].

Educational attainment was also an important demographic factor that influenced the subscale score of mental health. Patients with higher educational attainment had better mental health than those with lower educational attainment. It was possible that patients with higher educational attainment knew more about eye conditions, and sought suitable treatment before the visual impairment became too serious. Consistent with this possibility, Trillo et al. reported that a knowledge of eye conditions and educational attainment were important positive predictors of the quality of life [21]. Health education about cataracts would therefore improve the patient’s vision-related quality of life, especially mental health, so clinicians should provide general knowledge of cataracts when examining the patient’s eyes.

Overall, the CHI-NEI-VFQ-25 adequately assessed the vision-related quality of life for Chinese cataract patients. However, there were some questions that were not relevant for these patients. Questions related to driving had a very high non-response rate, and questions regarding ocular pain were not relevant for cataract patients, because most patients said they did not feel pain or discomfort in their eyes. Consistent with these findings, several studies reported that both the original English version and the Chinese version of the NEI-VFQ-25 were psychometrically flawed, and proposed modifications of the NEI-VFQ-25 by removing redundant and poor targeting questions [22–25]. Another limitation of this study was that over 93 % of the patients in this study were urban inhabitants. This study was performed in a city hospital where almost all the patients were urban inhabitants, while very few of the patients lived in rural areas.

## Conclusions

The results of the present study suggested that visual impairment due to age-related cataracts significantly decreased the vision-related quality of life. The BCVA in the better eye was the most important determinant associated with the decrease in vision-related quality of life. In addition, demographic factors, including sex, age, and educational attainment affected the quality of life of patients. Visual impairment was also negatively correlated with the mental health of the patients. Overall, this study provides information describing the vision-related quality of life in Chinese age-related cataract patients, which assists health care professionals in providing greater emphasis on cataract prevention and treatment.

## Abbreviations

CHI-NEI-VFQ-25: Chinese version of the National Eye Institute Visual Function Questionnaire-25
NEI-VFQ-25: National Eye Institute Visual Function Questionnaire-25
BCVA: Best corrected visual acuity
WHO: World Health Organization
SD: Standard deviation.
